# Potential Metabolite Nymphayol Isolated from Water Lily (*Nymphaea stellata*) Flower Inhibits MCF-7 Human Breast Cancer Cell Growth via Upregulation of Cdkn2a, pRb2, p53 and Downregulation of PCNA mRNA Expressions

**DOI:** 10.3390/metabo10070280

**Published:** 2020-07-08

**Authors:** Laila Naif Al-Harbi, Pandurangan Subash-Babu, Manal Abdulaziz Binobead, Maha Hussain Alhussain, Sahar Abdulaziz AlSedairy, Amal A Aloud, Ali A Alshatwi

**Affiliations:** Cancer Molecular Biology Research Lab, Department of Food Science and Nutrition, College of Food Science and Agriculture, King Saud University, Riyadh 11451, Saudi Arabia; lalharbi1@ksu.edu.sa (L.N.A.-H.); sbpandurangan@ksu.edu.sa (P.S.-B.); mbinobead@ksu.edu.sa (M.A.B.); mhussein@KSU.EDU.SA (M.H.A.); ssudairy@ksu.edu.sa (S.A.A.); aaloud@ksu.edu.sa (A.A.A.)

**Keywords:** *Nymphaea stellata*, MCF-7 cells, cytotoxicity, tumor suppressor gene, apoptosis

## Abstract

Controlled production of cyclin dependent kinases (CDK) and stabilization of tumor suppressor genes are the most important factors involved in preventing carcinogenesis. The present study aimed to explore the cyclin dependent apoptotic effect of nymphayol on breast cancer MCF-7 cells. In our previous study, we isolated the crystal from a chloroform extract of *Nymphaea stellata* flower petals and it was confirmed as nymphayol (17-(hexan-2-yl)-10,13-dimethylhexadecahydro-1*H*-cyclopenta[*a*]phenanthren-3-ol) using x-ray diffraction (XRD), Fourier transform infrared (FTIR), and mass spectroscopy (MS) methods. The cytotoxic effect of nymphayol on MCF-7 cells were analyzed using the 3-(4,5-dimethylthiazol-2yl)-2,5-diphenyl tetrazolium bromide (MTT) assay. The cellular and nuclear damage was determined using propidium iodide (PI) and acridine orange/ethidium bromide (AO/ErBr) staining. Tumor suppressor and apoptosis related mRNA transcript levels were determined using real-time polymerase chain reaction (RT-PCR). Nymphayol potentially inhibits MCF-7 cell viability up to 78%, and the IC_50_ value was observed as 2.8 µM in 24 h and 1.4 µM in 48 h. Treatment with nymphayol significantly increased reactive oxygen species (ROS) level and the tunnel assay confirmed DNA damage. We found characteristically 76% apoptotic cells and 9% necrotic cells in PI and AO/ErBr staining after 48 h treatment with 2.8 µM of nymphayol. Gene expression analysis confirmed significantly (*p* ≤ 0.001) increased mRNA levels of cyclin dependent kinase inhibitor 2A (Cdkn2a), retinoblastoma protein 2 (pRb2), p53, nuclear factor erythroid 2-factor 2 (Nrf2), caspase-3, and decreased B-cell lymphoma 2 (Bcl-2), murine double minute 2 (mdm2), and proliferating cell nuclear antigen (PCNA) expression after 48 h. Nymphayol effectively inhibited breast cancer cell viability, and is associated with early expression of Cdkn2a, pRb2, and activation of p53 and caspases.

## 1. Introduction

Incidence of cancer are recognized with altered apoptosis mechanism, genetic mutations, oxidative stress, hypoxia, and sustained intra cellular inflammation, while environmental factors are linked to ultraviolet ray exposure, radiation, and lifestyle [1]. Aberrant cellular mechanisms in the apoptotic signaling pathway results in uncontrolled cell progression, leading to carcinogenesis [2]. Apoptosis is highly sensitive and organized machinery, regulated by a set of sequential signal transduction genes and signaling proteins, which regulate the death receptor related extrinsic pathway and mitochondrial stress involved intrinsic pathway [3]. Therefore, stimulating or suppressing apoptotic factors such as tumor suppressor genes and cyclin dependent kinases aid in arresting cancer progression.

Tumor development is regulated by oncogenes, furthermore, tumor suppressor genes are activated or suppressed by epigenetic mechanisms [4]. The tumor suppressor p53 is stabilized and accumulated during uncontrolled cancer progression, but in stress or hyper proliferation signaling conditions, mdm2 negatively regulates p53, which leads to the transition from resting phase (G1) to DNA synthesis phase (S), and subsequently cancer cell progression continues. The cell-cycle gatekeeper gene p^14ARF^ neutralizing mdm2 function via cyclin dependent protein kinase (Cdkn2A) leads to increased levels of active p53 [5]. Targeting or early activation of CDKs and retinoblastoma (RB) resulting in the activation of p53 tumor-suppressor pathways is a fundamental requirement for the genesis of most human cancer treatment [6].

Plant sterols play a major role in cellular signaling and cell membrane organization, plant sterols are part of the cellular antioxidant effect and is also involved in hormone production [7]. Cells undergoing oxidative stress have revealed more cellular oxidative stress when compared to normal cancerous cells [7,8]. Plant sterols such as β-sitosterol and statin have been identified to increase antioxidant capacity and decreased NF-κB expression in fibroadenomas and breast cancer cells [9]. Despite significant advances in medical technology for the diagnosis and treatment of breast cancer, it still remains one of the most common devastating diseases and the second major cause of death worldwide [10].

**Figure 1:**
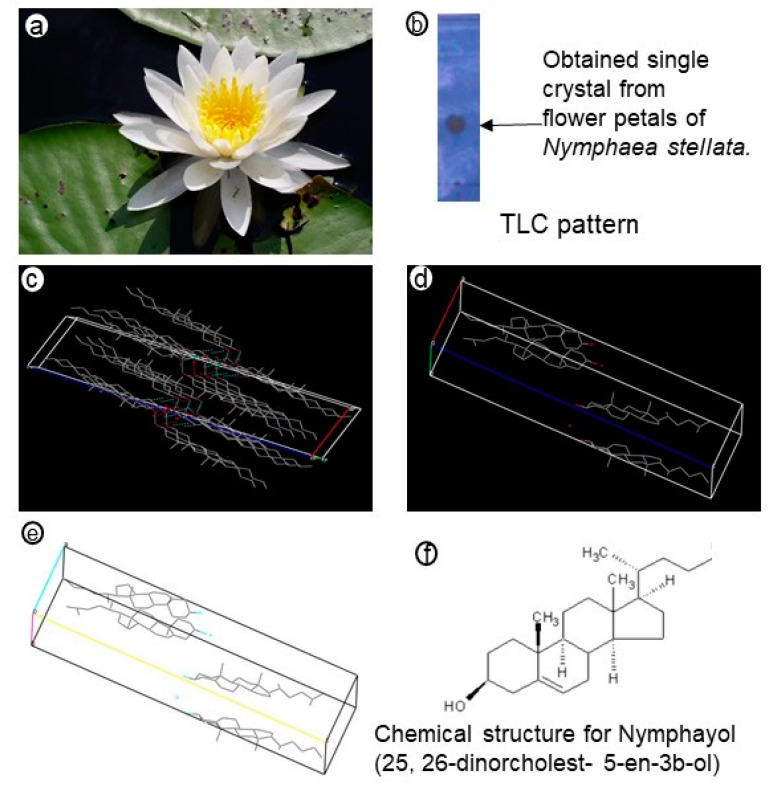
*Nymphaea stellata* flower petals (**a**) and thin layer chromatography (TLC) single spot of isolated crystal from the chloroform extract (**b**); (**c**–**e**) shows that the crystal structure refinement for the asymmetric unit consists of two molecules of 17-(hexan-2-yl)-10,13-dimethylhexadecahydro-1*H*-cyclopenta[*a*]phenanthren-3-ol and one water molecule; (**f**) shows the structure of the novel compound, 25,26-dinorcholest-5-en-3b-ol. The isolated novel crystal was named nymphayol.

The development of new anticancer agents for breast cancer is important to reduce the mortality caused by this disease. Nymphayol is a sterol terpenoid isolated from edible flower petals of the *Nymphaea stellata* Willd. flower (Figure 1a) [11]. *Nymphaea stellata* Willd. has been traditionally used to treat many diseases including cancer and possesses a broad range of pharmacological actions [12]. This plant exhibits different properties such as anti-inflammatory and anti-proliferative actions, suggesting that it could play a significant role as a novel suppressor for tumor genes. We have reported the antidiabetic and anti-inflammatory effect of nymphayol (25,26-dinorcholest-5-en-3b-ol) in animal and in vitro cell models [13,14]. The present study aimed to evaluate the potential of nymphayol on cytotoxicity, changes in cell and nuclear morphology, DNA damage, suppressor of tumor genes, and inducing early apoptosis in human MCF-7 breast cancer cells.

## 2. Results

In column chromatography, the *Nymphaea stellata* chloroform extract yielded a total of 74 fractions (each 150 mL); each fraction was spotted on a precoated Silica gel 60 F_254_, 0.25 mm thick thin layer chromatography (TLC) plate (Merck) and eluted in a hexane:ethyl acetate (4:1) system and fractions with similar Rf values in TLC pattern were pooled together. Finally, 17 major fractions were obtained and fraction 12 formed as a white colored amorphous crystal with traces of impurities. Fraction 12 eluted with hexane:ethyl acetate (30:70) yielded 4.8 gm. The crystal was washed with hexane and ethyl acetate to remove the impurities before finally 4.2 gm of pure crystal was obtained (melting point: 136 °C, literature melting point: 136–137 °C). The crystal tested positive in the Liebermann–Burchard test for sterols by appearing as a green color on treatment with acetic anhydride and con.H_2_SO_4._ It gave a single spot on TLC (Figure 1b) over silica gel (R_f_ = 0.7) with hexane:ethyl acetate (4:1) as the developing system.

X-ray diffraction (XRD) results provided the crystal data as (C_25_H_42_0)_2_. H_2_O (molecular formula), formula weight (*Mr*) 735.19, monoclinic, space group *P*2_1_ (No.4), the unit cell parameters obtained were a = 9.618(5), b = 7.518(5), c = 37.491(5) A^°^_,_ β = 94.483(5)^°^, *V* = 2703(2) A^3^, Z = 2, *Dc* = 0.903 Mg/m^3^, *F*(000) = 820, *µ*(M_0_Kα) = 0.054 mm^−1^, crystal size = 0.3 × 0.1 × 0.1 nm (Table 1; Figure 1c). The asymmetric unit consisted of two molecules of 17-(hexan-2-yl)-10,13-dimethylhexadecahydro-1*H*-cyclopenta[*a*]phenanthren-3-ol and one water molecule. Both molecules in the asymmetric unit were oriented in the same direction. The terminal methyl group of one of the molecule had disordered into two positions (Figure 1c). In addition, Figure 1d,e confirmed that two molecules were not conformationally identical, particularly at the terminal side chain. The molecules and their 2_1_ screw equivalents were linked through O-H…O (O_1_-H_1_A…O_2_, 2.06 Å, 167.9^0^; O_2_-H_2_…O_3_, 2.27 Å, 168.6^0^) hydrogen bonds to form hydrogen bonded dimmers. The packing of these dimmers in the lattice were stabilized through van der Waal’s interactions.

A search in the crystallographic database with the unit cell parameter revealed no hits related to this crystal’s unit cell parameter. The crystal was confirmed as a new crystal named nymphayol (25,26-dinorcholest-5-en-3b-ol [or] 17-(hexan-2-yl)-10,13-dimethylhexadecahydro-1*H*-cyclopenta[*a*]phenanthren-3-ol) and patented in Indian patent, 2007 [11]. In addition, the crystal was subjected to spectral analysis such as Fourier transform infrared spectroscopy (FT-IR) and mass spectrometry (MS) spectra analysis to reconfirm the functional group and molecular mass of the isolated compound (Supplementary Materials 1). The spectral data of FTIR [IR γ KBr/max cm^−1^: 3433 (hydroxyl); 2936, 2866, 1645 (trisub double bond); 1464, 1377, 1231, 1054, 801 (trisub double bond) (Supplementary Figure S1)] and MS [EIMS (*m*/*z*): 358 [m]^+^, 343 [m^+^-me], 273 [m-side chain]^+^, 325 [m–CH_3_–H_2_O], 287 [m^+^ -Ring B cleavage], 231 [m-side chain-ring D cleavage]^+^, 329 [m–CH_3_–CH_2_]^+^, 315 [m–CH_3_–(CH_2_)]^+^ (Supplementary Figure S2)) identified that the functional group and molecular mass (735.19, C_25_H_42_O) were similar to the crystallography data. The isolated single compound was confirmed as 25,26-dinorcholest-5-en-3b-ol and named as nymphayol (Supplementary Figure S3).

**Figure 2:**
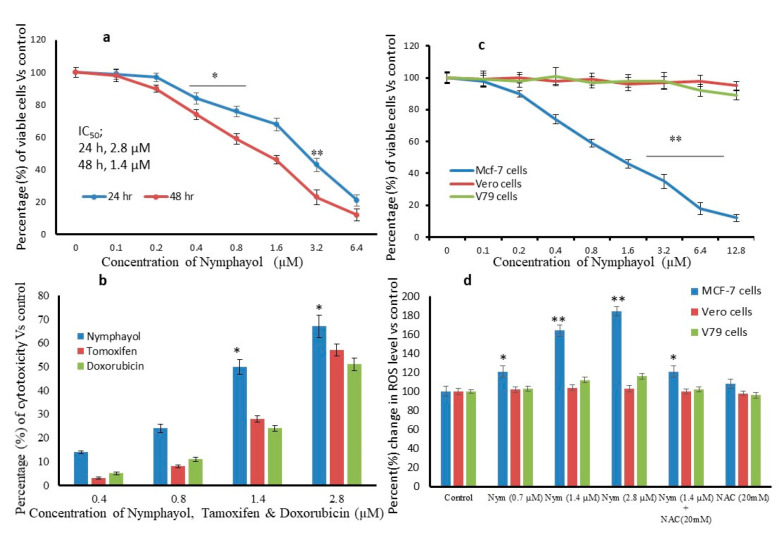
Cytotoxic effect of nymphayol on MCF-7 cells after 24 h and 48 h (**a**); (**b**) shows the comparative cytotoxic effect of nymphayol, tamoxifen, and fluorouracil after 48 h; (**c**) shows the cell specific cytotoxic effect between MCF-7 and normal (Vero and V79) cells after 48 h; (**d**) shows the comparative reactive oxygen species (ROS) production levels in vehicle control dimethyl sulfoxide (DMSO) and nymphayol treated MCF-7, Vero and V79 cells after 48 h. Data are expressed as the mean ± SD (n = 6). * *p* ≤ 0.01 vs. vehicle control, ** *p* ≤ 0.001 vs. vehicle control.

Figure 2a shows the inhibition of human breast cancer MCF-7 cell viability by nymphayol in a dose and time dependent manner. A total of 2.8 µM of nymphayol arrested 50% of MCF-7 cell growth in 24 h, and the same 50% arrest was achieved after 48 h with 1.4 µM of nymphayol. The IC_50_ dose, 1.4 µM and 2.8 µM dose of tamoxifen and doxorubicin inhibited only 35% and 46% of cell viability after 48 h (Figure 2b). Notably, nymphayol did not produce any significant cytotoxicity to noncancerous Vero and V79 cells at the selected higher concentration until 48 h (Figure 2c).

The level of intracellular reactive oxygen species (ROS) with the potential to initiate oxidative stress and DNA damage may contribute to cell cycle arrest or cellular apoptosis. In our study, ROS generation was found to be at the basal level in dimethyl sulfoxide (DMSO) (vehicle control) treated cancer (MCF-7) and noncancerous (Vero and V79) cells. Furthermore, 0.7, 1.4, and 2.8 µM concentrations of nymphayol treated to MCF-7, Vero, and V79 cells for 48 h significantly (*p* ≤ 0.001) increased ROS level only in MCF-7 cells. In MCF-7 cells, 23% of ROS production was observed even at the lower (0.7 µM) dose of nymphayol. However, Vero and V79 cells produced 3% and 16% of ROS in the tested higher concentration (2.8 µM) of nymphayol, respectively. A samples of 2.8 µM of nymphayol treated MCF-7 cells showed 84% increased ROS generation as reflected by increasing dichlorofluorescein (DCF) fluorescence after an incubation of 30 min compared to the vehicle control (Figure 2d). The cells pretreated with N-acetyl cysteine (NAC), an antioxidant, suppressed nymphayol-induced ROS generation (Figure 2d), which confirmed the nymphayol stimulated ROS production in MCF-7 cells.

**Figure 3:**
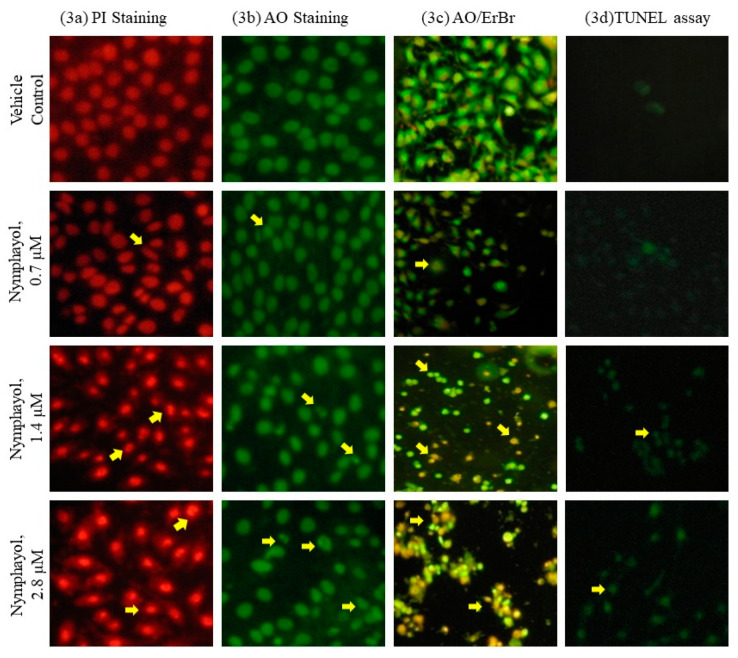
Propidium iodide (PI) (**a**), acridine orange (AO) staining (**b**), acridine orange/ethidium bromide (AO/ErBr)-staining (**c**) and terminal deoxynucleotidyl transferase dUTP nick end labeling (TUNEL) assay (**d**) images of vehicle control dimethyl sulfoxide (DMSO), 0.7 µM, 1.4 µM, and 2.8 µM of nymphayol treated MCF-7 cells after 48 h shown at 200x. In PI staining; 1.4 µM and 2.8 µM doses of nymphayol with a horse shoe shaped nucleus and shrunken nucleus confirmed the nuclear pyknosis and apoptosis. In AO/ErBr staining; 2.8 µM dose of nymphayol clearly showed proapoptotic (bright green), early apoptotic (light green) and late apoptotic (orange) cells.

Morphological changes of nymphayol treated MCF-7 cells after propidium iodide (PI) and acridine orange/ethidium bromide (AO/ErBr) staining were also investigated. PI and AO staining of 2.8 µM of nymphayol treated cells after 48 h showed abnormal nuclei, nuclei fragmentation, and horseshoe-shaped nucleus, which indicate apoptotic stimuli when compared to lower doses (Figure 3a,b). AO/ErBr staining of the MCF-7 cells showed distinguished pre, early, and late apoptotic cells via dark green, light green, and orange color (Figure 3c). The terminal deoxynucleotidyl transferase dUTP nick end labeling (TUNEL) assay confirmed the presence of MCF-7 cells in terminal apoptotic stages after nymphayol treatment (Figure 3d).

**Figure 4:**
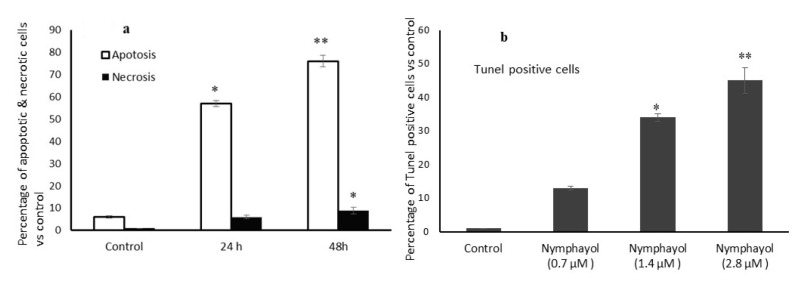
The percentage of apoptotic and necrotic cells after 48 h of nymphayol treatment is shown in (**a**). The TUNEL positive cells of nymphayol treated MCF-7 cells are shown in (**b**). Data are presented as the means ± SD (n = 6). Values sharing a common superscript as ** *p* ≤ 0.001 compared with the vehicle control, * *p* ≤ 0.01 compared with the vehicle control.

The manual counting of green florescence intensity indicates the degree of DNA damage induced by nymphayol. The cells stained with PI showed that 76% were apoptotic and 9% were in the necrotic stage (Figure 4a). In the terminal deoxynucleotidyl transferase dUTP nick end labeling (TUNEL) assay, nymphayol-treated MCF-7 cells clearly exhibited increased green florescence intensity (45%), which confirmed the presence of terminal DNA damage and apoptosis (Figure 4b).

**Figure 5:**
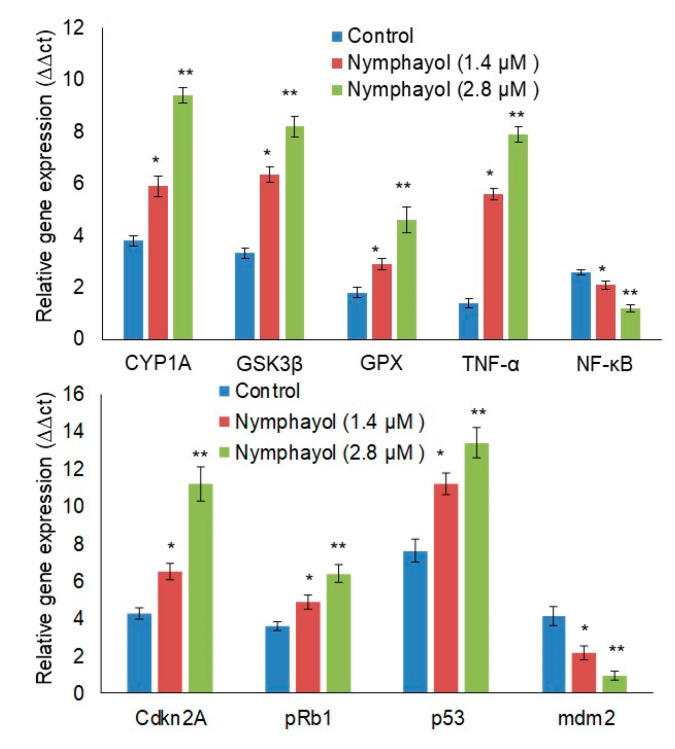
Effect of nymphayol on the expression levels of proapoptotic and tumor suppression related mRNA levels in MCF-7 cells after 48 h. Data are presented as the means ± SD (n = 6). Values sharing a common superscript as ** *p* ≤ 0.001 compared with 1.4 µM of nymphayol and vehicle control; * *p* ≤ 0.01 compared with the vehicle control.

The expression of metabolic oxidative stress-related genes such as CYP1A, GSK3β, GPX, TNF-α, and NF-κB in 1.4 and 2.8 µM of nymphayol-treated and vehicle control MCF-7 cells after 48 h is shown in Figure 5a. A higher dose of nymphayol showed significantly (*p* ≤ 0.001) higher gene expression of CYP1A (1.59-fold), GSK3β (1.29-fold), GPX (1.58-fold), TNF-α (1.41-fold), and decreased NF-ĸB (0.57-fold) when compared to a lower dose (1.4 µM) of nymphayol. Most notably, 1.4 µM of nymphayol treated MCF-7 cells showed significantly (*p* ≤ 0.01) higher expression levels of metabolic oxidative stress-related gene when compared to the vehicle control (DMSO).

The expression of Cdkn2A, pRb1, and p53 is shown in Figure 5b. There was a significant (*p* ≤ 0.001) increase in Cdkn2A (1.72 fold) and pRb1 (1.30), and a 1.19-fold increase in p53 after nymphayol treatment when compared to the lower dose. Most interestingly, the p53 expression was around 2-fold higher when compared to the vehicle control. In addition, the mRNA expression level of mdm2 was significantly decreased to 2.36-fold in the 2.8 µM dose when compared to the 1.4 µM dose of nymphayol.

**Figure 6:**
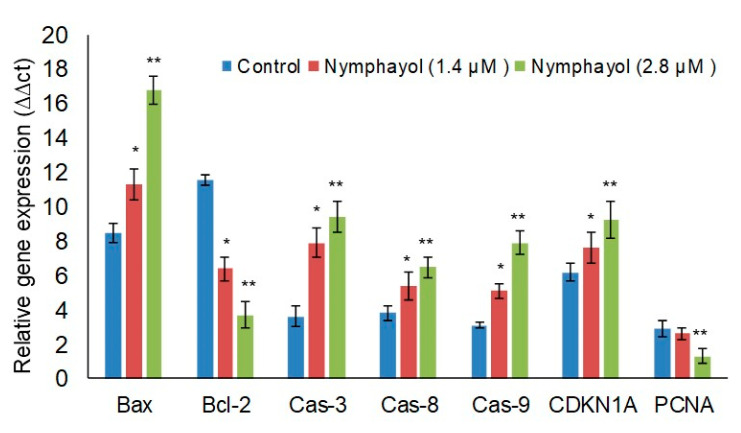
Effect of nymphayol on apoptosis related gene expression levels in MCF-7 cells after 48 h. Data presented as the means ± SD (n = 6). Values sharing a common superscript as ** *p* ≤ 0.001 compared with 1.4 µM of nymphayol and vehicle control; * *p* ≤ 0.01 compared with the vehicle control.

Figure 6 shows the changes in the mRNA levels of Bax, Bcl-2, caspases, CDKN1A, and PCNA expression in nymphayol treated and the vehicle control MCF-7 cells. In Figure 5, we observed a significant (*p* ≤ 0.001) increase in the mRNA levels of Bax (1.97-fold), caspase-3 (2.61-fold), caspase-8 (1.70-fold), caspase-9 (1.54-fold), and significant (*p* ≤ 0.001) decreases in Bcl-2 (3.13-fold) and PCNA (2.23-fold) expression in MCF-7 cells when compared with the vehicle control MCF-7 cells. The observed fold increase and decreased gene expression levels were calculated between the variation of the vehicle control (DMSO) and nymphayol treated MCF-7 cells.

## 3. Discussion

Nymphayol effectively inhibited breast cancer MCF-7 cell growth with the IC_50_ range of 2.8 μM in 24 h and 1.4 μM in 48 h compared to normal Vero and V79 cells. This finding confirmed the selective cytotoxicity of nymphayol against MCF-7 cells without causing toxicity to normal (V79 and Vero) cells. In this context, Min et al. [15] found that fucoidan induced cytotoxicity only in hepatocellular carcinoma, without causing senescence to normal Chang Liver cells. Moreover, the 2.8 μM (24 h, IC_50_) dose of nymphayol inhibited 92% of MCF-7 cells, but the same dose of tamoxifen and doxorubicin inhibited 35% and 46% of MCF-7 cells even after 48 h. The present finding was in line with previous reports such as *Nymphaea pubescens* [16] and phytosterols (sitosterol, campesterol, and stigmasterol) are well known for their anti-proliferative and anticancer activities [17].

Under physiological conditions, excessive accumulation of ROS plays a significant role in mediating cellular responses in cytotoxicity due to oxidative damage in hyper proliferative cancer cells [18]. Similarly, our previous reports demonstrated that at the molecular level, epoxy clerodane diterpene showed selective cytotoxicity via ROS-induced DNA damage in MCF-7 breast cancer cells [19]. In the present study, nymphayol increased ROS level and oxidative stress resulted in the initiation of cell, nuclear membrane damage, and DNA damage in MCF-7 cells was confirmed by the TUNEL assay. Recently, researchers have confirmed that the plant sterols (β-sitosterol, campesterol and stigmasterol) induced cytotoxicity in specific cancer cells via ROS stimulated signaling cascade involved apoptotic processes [17]. Fluorescent propidium iodide (PI) stain is permeable to the cell and nuclear membrane of damaged MCF-7 cells. PI staining of nymphayol treated MCF-7 cells showed chromatin condensation and horseshoe-shaped nuclei, and nuclear fragmentation confirmed the morphological changes related to those typical of apoptosis was observed using a fluorescent inverted microscope. The observed results suggest that induction of apoptosis by nymphayol was likely to be mediated by increased ROS production in hyper proliferating MCF-7 cells ending with oxidative stress. In this context, Yadav et al. [20] reported that oxidative stress mediated DNA damage associated with the activation of tumor suppressors and the mitochondria mediated caspase dependent apoptosis signaling pathway in MCF-7 cells.

The tumor suppressor p53 is a principal transcription factor regulating cellular pathways involved in apoptosis. In response to diverse stresses such as DNA damage, hypoxia, telomere shortening, oncogene, p53 is activated, leading to apoptosis induction [21]. Under normal cell physiology and unstressed cells, p53 is tightly regulated by murine double minute 2 (MDM2) by maintaining p53 at low levels. Thus, MDM2, a potent cellular antagonist of p53, limits p53 growth-suppressive function [22,23]. Our data showed upregulation of CDKN2A, pRb1, and p53 and downregulation of MDM2 in nymphayol-treated MCF-7 cells when compared with the control. A recent study showed that HepG2 cells treated with fucoidan induced apoptosis, which might be mediated by upregulating p16^(INK4a)^-Rb and p14^(Arf)^-p53 pathways [15]. Our results showed that nymphayol inhibits Bcl-2 expression, which plays a significant role in regulating cell proliferation and apoptosis [24]. Bcl-2 overexpression is observed in the majority of human cancers [25]. Anti-apoptotic family members such as Bcl-2 play a pivotal role in inhibiting apoptosis [26].

Caspases serve as primary effectors during the process of the apoptosis signaling pathway [18,27]. In response to apoptotic signals, caspases are rapidly activated [28]. Activation of caspases, in particular caspase 3 and 9, cleaves poly (ADP-ribose) polymerase-1 (PARP-1), leading to cell apoptosis. PARP has received considerable attention for use as a main target for many chemotherapeutic drugs, suggesting the significant role of PARP in maintaining genomic stability and repairing DNA [29]. Cyclin-dependent kinase inhibitor 1A (CDKN1A or p21) carries two functional domains, allowing them to bind to PCNA and Cdk/cyclins [30]. CDKN1A inhibits PCNA-dependent DNA replication by preventing PCNA from contributing to DNA polymerase δ and ε function. In addition, it induces cell growth arrest after DNA damage [30]. Consistent with the above findings, in our study, nymphayol treatment significantly increased the mRNA expression levels of caspase-3, caspase-8, caspase-9, and PARP and downregulated PCNA.

Overall, the cytotoxic effect of nymphayol on breast cancer MCF-7 cells and its possible mechanism of action were explored. We found that nymphayol increased ROS generation and stimulated hyper proliferative signals in MCF-7 cells, which effectively increased the early expression of cyclin dependent kinase (CDKN2A), aiding in the accumulation of active tumor suppressor p53. Furthermore, active p53 stimulated caspase dependent mitochondria mediated apoptotic signaling pathway related genes. This resulted in upregulated levels of caspase 3, caspase 9, CDKN1A expressions, and downregulated PCNA expressions was associated with cell growth arrest. Further studies are needed to confirm the anticancer mechanistic effect of nymphayol using Annexin V-FITC based apoptotic and necrotic cell sorting and western blot based protein quantification using in vitro and in vivo models.

## 4. Materials and Methods

### 4.1. Cell Culture Materials

Propidium iodide (PI), acridine orange (AO), and ethidium bromide (ErBr) were procured from Sigma-Aldrich (St. Louis, MO, USA). The DeadEnd Terminal deoxynucleotidyl transferase dUTP Nick End Labeling (TUNEL) Assay Kit was procured from Promega (Madison, WI, USA). The QuantiTect Primer Assay, Fast Lane Cell cDNA Kit, and QuantiFast SYBR Green PCR Kit were procured from Qiagen (Hilden, Germany). All other chemicals used in this study were cell culture grade.

### 4.2. N. Stellata Flower Chloroform Extract (NSFCExt) Preparation

The fresh petals of *N. stellate* flower were collected, shade dried, coarsely powered, and used for extraction. A total of 500 gm of dry petal powder was kept in an aspirator bottle; 1.5 L of chloroform was used, and the mixture was kept in a shaker (200 rpm) for 48 h. Then, the liquid portion containing the extract was filtered using Whatman filter paper (no. 2) on a Buchner funnel. This procedure was repeated three times and all extracts were decanted and combined. The solvent was removed by vacuum distillation in a rotary evaporator at 60 °C and the extracts were placed in pre-weighed flasks before drying.

### 4.3. Isolation of Crystal by Column Chromatography

Chloroform extract (10 gm) was adsorbed on silica gel (Acmae’s 60–120 mesh) and chromatographed on a silica gel (Acmae’s 100–200 mesh) column initially eluted with a continuous suitable system and gradually increasing the polarity of the mixture of solvents [hexane (non-polar) to methanol (polar)]. Eluted fractions were evaluated using TLC and a similar TLC patterns were pooled into major fractions.

### 4.4. Crystal Structure Refinement Using X-Ray Diffraction Method

The x-ray data of the crystal was recorded using an Enraf Nonious CAD4 X-ray and BRUKER AXS Kappa Apex 2 diffractometer. XRD analysis was carried out at the Indian Institute of Technology-Madras, Chennai. A crystal of suitable size (0.3 × 0.1 × 0.1 mm^3^) was inspected for single crystallinity using a LEICA DMLSP polarizing microscope and mounted on a Kappa Apex2, CCD diffractometer equipped with graphite monocromated Mo (Kα) radiation, (λ = 0.71073 Å). The unit cell parameters were obtained using reflections scanned from three different zones of the reciprocal lattice. The intensity data were collected using ω and φ scan with a frame width of 0.5°. The frame integration and data reduction were performed using Bruker SAINT-Plus (Version 7.06a) software. Multiscan absorption corrections were applied to the data using SADABS (Bruker axs) software (this data published in an Indian patent, 2007) [11,13].

### 4.5. Cell Culture

The MCF-7 human breast cancer cell line was obtained as a gift from Mahatma Gandhi-Doerenkamp Center (MGDC), National Center for Alternatives to Animal Experiments (NCAAE), Bharathidasan University, India. Vero and V79 hamster lung fibroblast cell lines were obtained from the National Center for Cell Sciences (NCCS), Pune, India. RPMI-1640 supplemented with 10% (*v*/*v*) heat inactivated Fetal Bovine Serum (FBS), 2 mM l-glutamine, 100 U/mL of penicillin, and 100 μg/mL of streptomycin was used to culture the cells at 37 °C in a humidified atmosphere of 5% CO_2_ (Thermo Scientific, Waltham, MA, USA).

### 4.6. In Vitro Cytotoxicity Assay Using 3-(4,5-Dimethylthiazol-2yl)-2,5-Diphenyl Tetrazolium Bromide (MTT)

MCF-7 cells were seeded (1 × 10^4^ cells/mL) in a 96-well culture plate and cultured for 24 h before treatment. Vero cells and V79 hamster lung fibroblast were also cultured using the same protocol. After 24 h, nymphayol was treated with increasing concentrations (0, 0.1, 0.2, 0.4, 0.8, 1.6, 3.2, and 6.4 µM) and continued to incubate for the next 24 h and 48 h, respectively. Doxorubicin and tamoxifen were used as the positive control. After incubation, 20 μL of mitochondrial dehydrogenase enzyme specific dye MTT (1 mg/mL) was added to the treated cells and incubated in the dark for 4 h at 37 °C. The reaction of MTT with viable cell mitochondrial dehydrogenase and produced purple formazan crystals were dissolved using 100 µL DMSO [31]. Then, the plates were absorbed under 492 nm using a micro plate reader.

### 4.7. Measurement of Intracellular Reactive Oxygen Species (ROS)

Cellular reactive oxygen species (ROS) was quantified using 2′,7′-dichlorofluorescin diacetate (DCFH-DA) [32]. MCF-7 cells were cultured in a 24-well plate and treated with 0.7, 1.4, and 2.8 µM nymphayol. In addition, MCF-7 cells were treated with the positive control of 20 mM N-acetyl cysteine. After 48 h incubation, the cells were washed twice with HBSS and then incubated in 2 mL of the DCFH-DA working solution at 37 °C for 30 min. The stable compound DCFH-DA diffuses into the cell, then hydrolyzes to form DCHF by the action of intracellular esterase. The presence of hydrogen peroxide or low molecular-weight peroxides present in cell oxidizes DCHF to the highly fluorescent green colored 2V,7V-dichlorofluorescein (DCF) compound. The fluorescent green color was measured using a SpectraMax Gemini XS fluorometer (Molecular Devices, Cambridge Scientific, Watertown, MA, USA) with an excitation wavelength of 485 nm and an emission wavelength of 520 nm.

### 4.8. Apoptosis Related Cellular and Nuclear Morphology Analysis

Cellular morphology for characteristic apoptotic and necrotic morphological changes after nymphayol treatment were determined using propidium iodide (PI) and acridine Orange/ethidium bromide (AO/ErBr) described by Leite et al. [33]. Briefly, MCF-7 cells were treated with nymphayol at 0.7, 1.4, and 2.8 µM for 48 h in 24 well plates. After incubation, cells were rinsed with PBS and stained with 500 µL of PI (1 mg/mL) or AO:ErBr (1:1.4 mg/mL) solutions. Within a few seconds of staining, cells were gently rinsed with PBS and images were captured using an inverted fluorescent microscope (Carl Zeiss, Jena, Germany) fitted with a 530/620 nm filter and observed at 200× magnification. The percentage of apoptotic and necrotic cells were determined by using a random sample of 300 stained cells, examined under inverted fluorescence microscope, and the pathological changes was counted manually.

### 4.9. Terminal Deoxynucleotidyl Transferase (TdT)-Mediated dUTP Nick End Labeling (TUNEL) Assay

Cells were allowed to grow on cover slips and treated with nymphayol at 0.7, 1.4 and 2.8 µM for 48 h along with a control. After 48 h, the cells were fixed with 4% paraformaldehyde, and DNA fragmentation was observed using terminal deoxynucleotidyl transferase (TdT)-mediated dUTP-digoxigenin nick-end labeling technique as per the manufacturer’s protocol. The results were presented as a representation from a series of three separate experiments.

### 4.10. Quantitative Real-Time Polymerase Chain Reaction (RT-PCR) Analysis of Tumor Suppressor, Oxidative Stress, and Apoptosis Related Genes

The cDNA was directly prepared from nymphayol treated cells using a Fastlane® Cell cDNA kit (QIAGEN, Hilden, Germany) after 48 h. The mRNA expression levels of oxidative stress-related genes [cytochrome P450 1A (CYP1A)], glutathione peroxidase (GPx), glutathione synthase kinase 3 beta (GSK3β), tissue necrotic factor–alpha (TNF-α), and nuclear factor kappa B (NF-κB), tumor suppressor genes [cyclin dependent kinase inhibitor 2A (Cdkn2A), retinoblastoma protein 2 (pRb1), p53, and murine double minute 2 (mdm2)], apoptotic genes (B-cell associated X (Bax), B-cell lymphoma-2 (Bcl-2), cyclin dependent kinase inhibitor 1A (Cdkn1A), caspases, and proliferating cell nuclear antigen (PCNA) as well as the reference gene glyceraldehyde 3-phosphate dehydrogenase (GAPDH) were assayed using gene-specific SYBR Green-based QuantiTect® primer assays (QIAGEN, Germany). Primer sequences for the antioxidant and apoptosis related genes are provided in Table 2. The gene expression level was then calculated as previously described by Yuan et al. [34]. To determine the relative expression levels, the following formula was used: ΔΔCt (comparative threshold) = ΔCt (Treated) − ΔCt (Control).

### 4.11. Statistical Analysis 

SPSS/11.5 software was used for the statistical significance evaluation. The values were analyzed using one-way analysis of variance (ANOVA) followed by Tukey’s test [35]. All results were four replicates in each group (mean ± SD) and the differences were presented statistically significant at *p* ≤ 0.01 and *p* ≤ 0.001.

## 5. Conclusions

Nymphayol possesses potent anti-proliferative effects in breast cancer MCF-7 cells. Nymphayol, a sterol triterpenoid, has been found to induce apoptosis in human breast cancer cells by the modulation of mitochondria mediated pathways involved and the activation of caspases linked with the apoptotic mechanism. The mechanistic anticancer effects were associated with the early expression of CDKN2A and pRb2 and activation of p53 and caspases. High expression of CDKN2A arrest p53–mdm2 amalgamation led to a higher availability of active p53. The sterol triterpenoid, nymphayol may play a complementary role against breast cancer therapy or synergistic role with currently used anticancer drugs for chemoprevention.

## Figures and Tables

**Table 1:** Crystal data and structure refinement for the novel compound, nymphayol, isolated from the chloroform extract of *Nymphaea stellata*.

Empirical formula	C_50_ H_86_ O_3_
Formula weight	735.19
Temperature	293(2) K
Wavelength	0.71073 A
Crystal system, space group	Monoclinic, P21
Unit cell dimensions	a = 9.618(5) A alpha = 90.000(5) deg. b = 7.518(5) A beta = 94.483(5) deg. c = 37.491(5) A gamma = 90.000(5) deg.
Volume	2703(2) A^3^
Z, Calculated density	2, 0.903 Mg/m^3^
Absorption coefficient	0.054 mm^−1^
F(000)	820
Crystal size	0.3 × 0.1 × 0.1 mm
Theta range for data collection	2.12 to 20.45 deg.
Limiting indices	−9 ≤ h ≤ 9, −7 ≤ k ≤ 7, −36 ≤ l ≤ 36
Reflections collected/unique	16593/5337 [R(int) = 0.0406]
Completeness to theta = 20.45	99.2%
Absorption correction	Semi-empirical from equivalents
Max. and min. transmission	0.9560 and 0.9140
Refinement method	Full-matrix least-squares on F^2^
Data/restraints/parameters	5337/11/484
Goodness-of-fit on F^2	1.105
Final R indices [I > 2sigma(I)]	R1 = 0.0842, wR2 = 0.2505
R indices (all data)	R1 = 0.0991, wR2 = 0.2687
Absolute structure parameter	−2(4)
Extinction coefficient	0.0013(17)
Largest diff. peak and hole	0.404 and −0.218 e.A^−3^

**Table 2:** Primers used in the Sybrgreen based real-time polymerase chain reaction (RT-PCR).

Primer	Forward Sequence (5’ to 3’)	Reverse Sequence (5’ to 3’)
CYP1A1	GCTGACTTCATCCCTATTCTTCG	TTTTGTAGTGCTCCTTGACCATCT
GSK3β	GGAACTCCAACAAGGGAGCA	TTCGGGGTCGGAAGACCTT
GPX	GTGCTCGGCTTCCCGTGCAAC	CTCGAAGAGCATGAAGTTGGGC
TNF-α	CTCTTCTGCCTGCTGCACTTTG	ATGGGCTACAGGCTTGTCACTC
NF-κB	GCGCTTCTCTGCCTTCCTTA	TCTTCAGGTTTGATGCCCCC
CDKN2A	CCTTCCAATGACTCCCTCC	TCAGAAACCCTAGTTCAAAGGA
pRb1	CTCGTGCTGATGCTACTGAGGA	GGTCGGCGCAGTTGGGCTCC
p53	CCTCAGCATCTTATCCGAGTGG	TGGATGGTGGTACAGTCAGAGC
mdm2	CCCAAGACAAAGAAGAGAGTGTGG	CTGGGCAGGGCTTATTCCTTTTCT
Bax	TCAGGATGCGTCCACCAAGAAG	TGTGTCCACGGCGGCAATCATC
Bcl-2	GTGGATGACTGAGTACCT	CCAGGAGAAATCAAACAGAG
Caspase-3	ACATGGAAGCGAATCAATGGACTC	AAGGACTCAAATTCTGTTGCCACC
Caspase-8	CCGCAAAGGAAGCAAGAA	GGTAGGTAATCAGCAAATCCAGT
Caspase- 9	GCTCTTCCTTTGTTCATC	CTCTTCCTCCACTGTTCA
CDKN1A	AGGTGGACCTGGAGACTCTCAG	TCCTCTTGGAGAAGATCAGCCG
PCNA	CAAGTAATGTCGATAAAGAGGAGG	GTGTCACCGTTGAAGAGAGTGG
GAPDH	GTCTCCTCTGACTTCAACAGCG	ACCACCCTGTTGCTGTAGCCAA

## Supplementary Materials

The following are available online at https://www.mdpi.com/2218-1989/10/7/280/s1, Figure S1: FT-IR spectrum of single compound, Figure S2: MS spectrum of single compound, Figure S3: Structure of isolated compound 26,27-dinorcholest-5-en-3-β-ol (It was named as nymphayol).
