# Supplementary material for: Potential Metabolite Nymphayol Isolated from Water Lily (Nymphaea stellata) Flower Inhibits MCF-7 Human Breast Cancer Cell Growth via Upregulation of Cdkn2a, pRb2, p53 and Downregulation of PCNA mRNA Expressions
Source: Metabolites. 2020 Jul 8;10(7):280. doi: 10.3390/metabo10070280 (PMC7408605; doi:10.3390/metabo10070280)
Supplement: Supplementary file 1 [file metabolites-10-00280-s001.pdf]

Supplementary data 1

Schematic representation for extraction, isolation and spectral analysis for isolated single crystal from *Nymphaea stellata* Wild. Chloroform extract

S.1. Methods

S.1.1. Isolation of Single Crystal from *Nymphaea stellata*-Flower Petals Chloroform Extract.

Schematic representations for Extraction, isolation and identification of isolated compound from *Nymphaea stellata*-flower chloroform extract.

***Nymphaea stellata* flower petals**- Flower (500 gm, dry & coarsely powdered)

↓

Sequential Extraction with organic solvents

Hexane | Chloroform | Ethyl acetate | Methanol | Aqueous

↓ *(Active)*

Column chromatography

↓ *Elution of compounds with increasing polarity of Hexane, Hexane: Chloroform and Hexane: Ethyl acetate*

74 fractions were obtained

↓ *Based on the TLC profiles, fractions were pooled together and 17major fractions were obtained*

Fr 12, crystal

All the fractions were screened for anticancer effect

↓

Fraction 12 shown significant glycemic control and it is formed as crystal.

↓

Subjected to XRD and spectral analysis

↓

Single crystal (COMPOUND)

S.1.2. Spectral analysis

We have isolated a single crystal from *Nymphaea stellata* Willd. Chloroform extract. The crystal was subjected to XRD analysis and the structure was refined as nymphayol (25, 26-dinorcholest- 5-en-3b-ol [or] 17-(hexan-2-yl)-10,13-dimethylhexadecahydro-1*H*-cyclopenta[*a*] phenanthren-3-ol) and its molecular formula was confirmed as $C_{25}H_{42}O$). H_2O. In addition, the crystal was subjected to spectral analysis like, FT-IR and MS spectra analysis to reconfirm the functional group and molecular formula of isolated compound. FT-IR spectra were obtained in Shimadszu by KBr pellet method. IR spectra were analyzed using Perkin Elmer (FT-IR) spectrophotometer. High resolution Elecron Impact Mass Spectroscopy (EI-MS) was performed. Mass spectra were obtained using Jeol JMS-DX30 spectrometer.

S.2. Results

S.2.1. FT- IR

IR γ KBr/max cm^{-1}: 3433 (hydroxyl); 2936, 2866, 1645 (trisub double bond); 1464, 1377, 1231, 1054, 801 (trisub double bond) (Figure S.1).

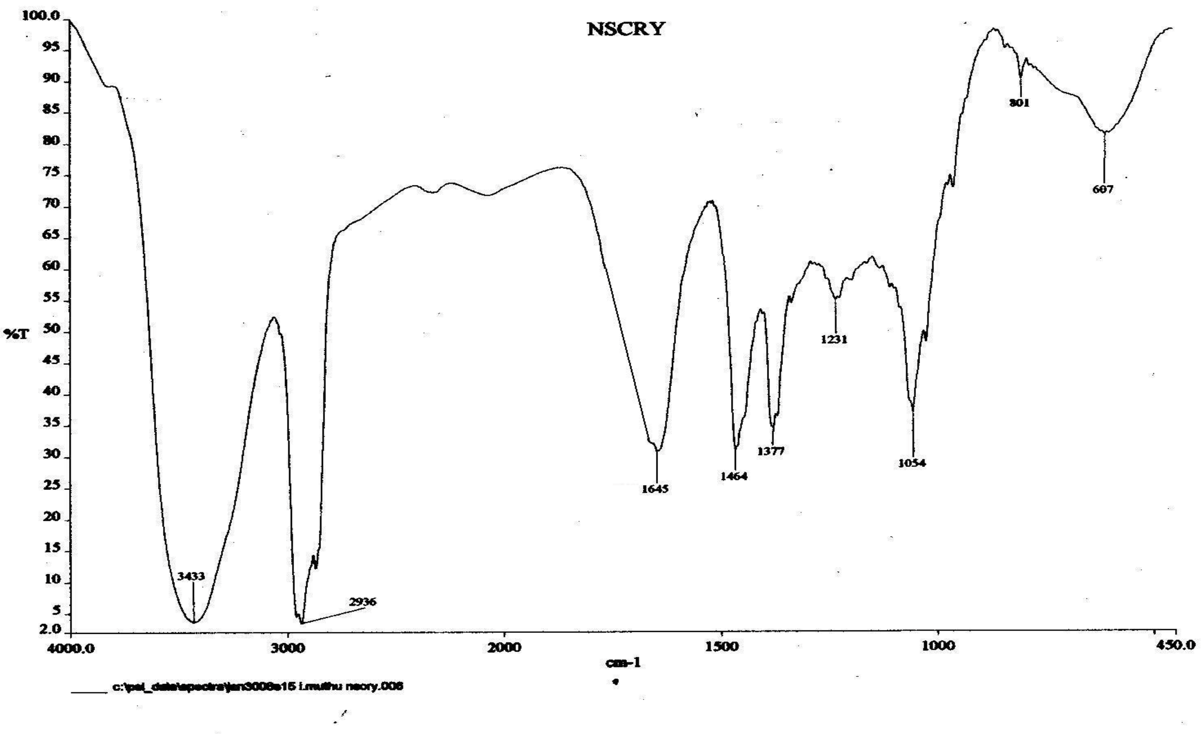

Figure S1. FT-IR spectrum of single compound.

S.2.2. MS

EIMS (m/z): 358 $[m]^+$, 343 $[m^+$-me], 273 [m-side chain]$^+$, 325 [m-CH_3-H_2O], 287 $[m^+$-Ring B clevage], 231 [m-side chain-ring D cleavage]$^+$, 329 [m-CH_3-CH_2]$^+$, 315 [m-CH_3-(CH_2)]$^+$ (Figure S.2). Molecular formula is $C_{25}H_{42}O$ and formula weight is 735.19.

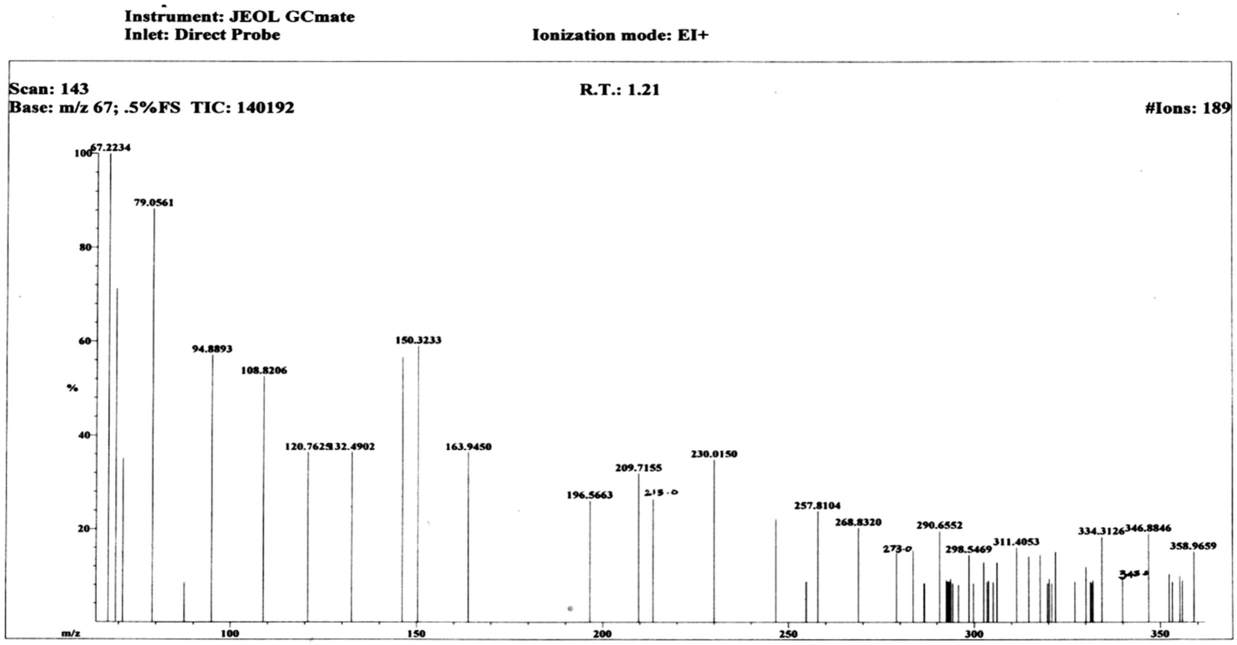

Figure S2. MS spectrum of single compound.

S.3. Discussion

The FT-IR and MASS spectral data of the isolated single compound have been identified with the functional group and molecular mass (735.19, $C_{25}H_{42}O$) were similar with the crystallography data (Supplementary Figure S -3). The spectral data were comparable with those of similar reported values by Carlson et al., (1978) [1]. In our previous study, bioassay guided extraction, fractionation, isolation of *N. Stellata* flower chloroform extract and spectral characterization of 26, 27-dinorcholest-5-en-3-β-ol (Nymphayol) have been well documented [2].

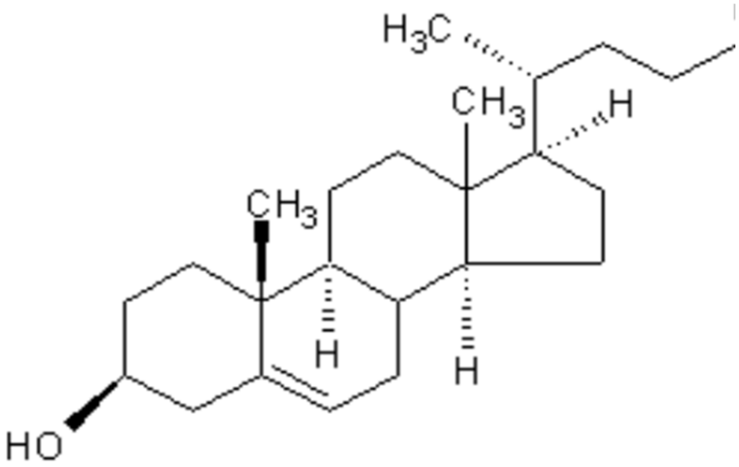

Figure S3. Structure of isolated compound 26, 27-dinorcholest-5-en-3-β-ol (It was named as nymphayol).

S.4. References

1. Carlson, R.M.K.; Popov, S.; Massey, I.; Delseth, C.; Ayanoglu, E.; Varkony, T.H.; Djerassi, C. Minor and trace sterols in marine invertebrates. VI. Occurrence and possible origins of sterol possessing unusually short hydrocarbon side chain. Bioorganic chemistry 1978, 7, 453-479.
2. Subash-Babu, P.; Ignacimuthu, S.; Agastian, P.; Varghese, B. Partial regeneration of beta-cells in the islets of Langerhans by Nymphayol a sterol isolated from Nymphaea stellata (Willd.) flowers. Bioorg. Med. Chem. 2009, 17(7), 2864-70.
